# Compressive Strength Estimation of Geopolymer Composites through Novel Computational Approaches

**DOI:** 10.3390/polym14102128

**Published:** 2022-05-23

**Authors:** Muhammad Nasir Amin, Kaffayatullah Khan, Waqas Ahmad, Muhammad Faisal Javed, Hisham Jahangir Qureshi, Muhammad Umair Saleem, Muhammad Ghulam Qadir, Muhammad Iftikhar Faraz

**Affiliations:** 1 Department of Civil and Environmental Engineering, College of Engineering, King Faisal University, Al-Ahsa 31982, Saudi Arabia; kkhan@kfu.edu.sa (K.K.); hqureshi@kfu.edu.sa (H.J.Q.); 2 Department of Civil Engineering, COMSATS University Islamabad, Abbottabad 22060, Pakistan; waqasahmad@cuiatd.edu.pk (W.A.); arbabfaisal@cuiatd.edu.pk (M.F.J.); 3 Service Stream Limited Co., Chatswood, NSW 206, Australia; umair.saleem@servicestream.com.au; 4 Department of Environmental Sciences, COMSATS University Islamabad, Abbottabad 22060, Pakistan; hashir785@gmail.com; 5 Department of Mechanical Engineering, College of Engineering, King Faisal University, Al-Ahsa 31982, Saudi Arabia; mfaraz@kfu.edu.sa

**Keywords:** machine learning, geopolymer concrete, artificial intelligence, prediction models, compressive strength

## Abstract

The application of artificial intelligence approaches like machine learning (ML) to forecast material properties is an effective strategy to reduce multiple trials during experimentation. This study performed ML modeling on 481 mixes of geopolymer concrete with nine input variables, including curing time, curing temperature, specimen age, alkali/fly ash ratio, Na_2_SiO_3_/NaOH ratio, NaOH molarity, aggregate volume, superplasticizer, and water, with CS as the output variable. Four types of ML models were employed to anticipate the compressive strength of geopolymer concrete, and their performance was compared to find out the most accurate ML model. Two individual ML techniques, support vector machine and multi-layer perceptron neural network, and two ensembled ML methods, AdaBoost regressor and random forest, were employed to achieve the study’s aims. The performance of all models was confirmed using statistical analysis, k-fold evaluation, and correlation coefficient (R^2^). Moreover, the divergence of the estimated outcomes from those of the experimental results was noted to check the accuracy of the models. It was discovered that ensembled ML models estimated the compressive strength of the geopolymer concrete with higher precision than individual ML models, with random forest having the highest accuracy. Using these computational strategies will accelerate the application of construction materials by decreasing the experimental efforts.

## 1. Introduction

Construction is a vital component of any economy [1]. The building sector generates large amounts of waste and emits considerable amounts of greenhouse gases (GHGs) into the environment [2,3]. Cement-based materials such as concrete are the primary building materials utilized in the construction industry worldwide [4,5,6,7]. It is now well accepted that the manufacture of cement leads to the emission of significant amounts of GHGs that contribute to global warming, as well as the use of significant amounts of raw materials [8,9,10]. It has been calculated that around two tons of raw materials (shale and limestone) are consumed in the manufacturing of one ton of cement, and approximately one ton of carbon dioxide (CO_2_) and nitrogen oxide (NO) pollutants are released [11]. With over two billion tons of GHGs emitted yearly as a result of cement manufacture, cement production accounts for approximately 6% of global anthropogenic GHG emissions [12,13,14]. The extensive use of natural raw materials in the manufacture of cement has also resulted in the overexploitation of natural resource reserves, resulting in a degradation of the aesthetics of the environment and the modification of ecosystems [15,16]. Apart from the substantial GHG emissions associated with cement manufacture, the process is extremely energy demanding [17,18]. Recent urbanization, particularly in developing nations, has exacerbated the negative environmental effect of cement manufacturing [19]. As a result, it is critical that sustainable alternatives to cement be utilized in building applications in order to preserve the environment’s sustainability [20,21]. Numerous waste products created by various sectors can be utilized as sustainable substitutes for the traditional resources used in the cement manufacturing process. As a result, the utilization of such wastes in the manufacturing of a sustainable alternative to cement would result in a considerable decrease in GHG emissions, the cost of raw materials, and the use of natural raw resources connected with cement [22]. Materials that have been activated with alkali, such as geopolymers, may be preferred to conventional cement concrete [23,24,25].

Davidovits was the first to propose geopolymers consisting of semi-crystalline three-dimensional aluminosilicate materials in 1979 [26]. These geopolymers may be manufactured using a variety of source materials, including fly ash, metakaolin, ground granulated blast furnace slag, and rice husk bark ash [27,28,29,30]. Since then, scientists have paid close attention to geopolymers due to their unique combination of superior mechanical performance, chemical and fire resistance, low CO_2_ emissions, and low energy consumption [31,32]. These features are intimately connected to the chemical interactions between aluminosilicate and alkali-polysialate [33]. The use of geopolymer concrete (GeoPC) in place of conventional cement concrete results in an embodied carbon reduction of up to 80%, depending on the precursor and activator utilized [34]. GeoPC is mostly composed of waste materials from various industrial and agricultural activities. GeoPC may be considered more ecologically friendly and an efficient method of managing enormous amounts of waste created by industries [35,36,37]. The utilization of locally accessible materials as precursors, such as laterite soil, can help increase the sustainability of GeoPCs [11]. Thus, by utilizing geopolymers as a sustainable alternative to cement, GHG emissions, raw material consumption, and waste management costs would be significantly reduced [38,39,40].

The practice of developing models for forecasting the strength of concrete is ongoing in order to reduce unnecessary test repetitions and material waste. There are several prominent models for modeling concrete properties, such as best fit curves (based on regression analysis). However, due to the nonlinear behavior of concrete [41], regression models generated using this technique may not accurately represent the underlying nature of the material. Additionally, regression methods may understate the effect of constituent materials in concrete [42]. Artificial intelligence techniques such as machine learning (ML) are some of the more contemporary modeling techniques that have been used in the area of civil engineering. These approaches use input parameters to model responses, and the output models are validated by experimentation. For construction applications, ML algorithms estimate concrete strength [43,44,45,46,47], bituminous mixture performance [48], and concrete durability [49,50,51].

This study focuses on the application of ML techniques to forecast the compressive strength (C-S) of GeoPC. Four distinct ML techniques were used, including support vector machine (SVM), multi-layer perceptron neural network (MLPNN), AdaBoost regressor (AR), and random forest (RF) to anticipate the C-S of GeoPC. The effectiveness of all techniques was evaluated by applying statistical tests and correlation coefficients (R^2^). Furthermore, k-fold analysis and error distributions were used to determine the validity of each technique. SVM and MLPNN are individual ML techniques, while AR and RF are ensemble ML methods [52]. This study is interesting in that it predicts the C-S of GeoPC utilizing both individual and ensemble ML techniques. However, experimental studies require considerable human effort, the cost for experimentation, and time for material collection, sample casting, curing, and testing. The application of novel methods, such as ML, in the construction field to anticipate material characteristics will decrease the aforesaid issues by obviating the need for experimental work. ML methods need a data set, which may be collected from the past studies since a considerable amount of investigation has been undertaken to determine material characteristics, and the data set might be utilized for training the ML models and forecasting the material properties. The purpose of this work is to ascertain the top appropriate ML method for the C-S estimation of GeoPC based on the results estimation and the effect of input variables on ML model performance.

## 2. Data Description

**Figure 1:**
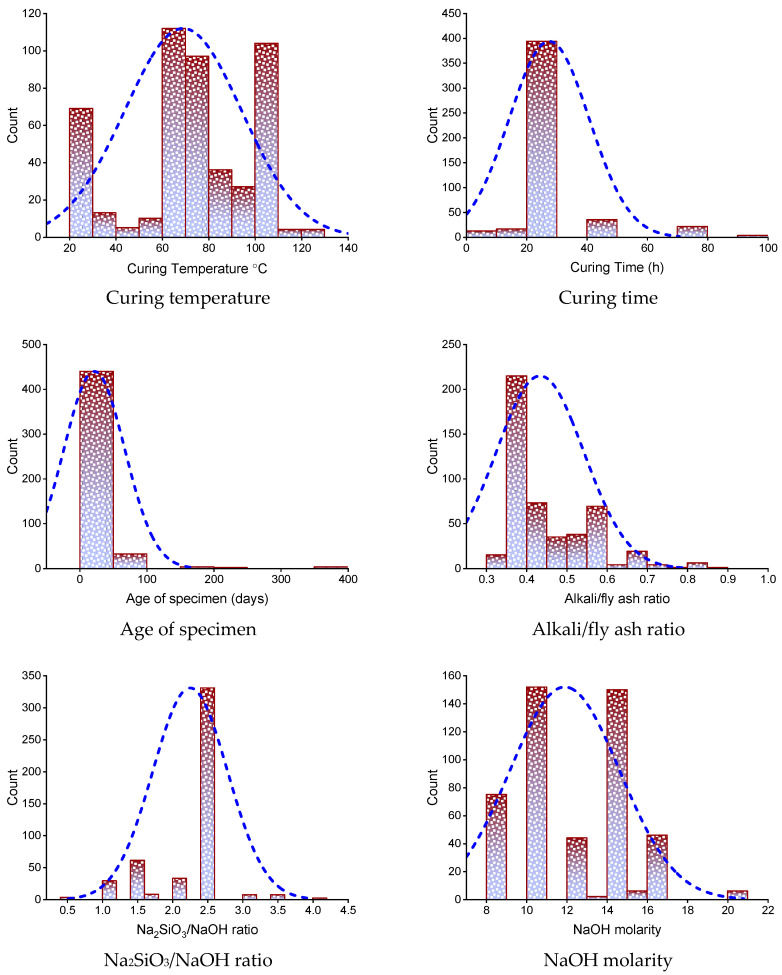

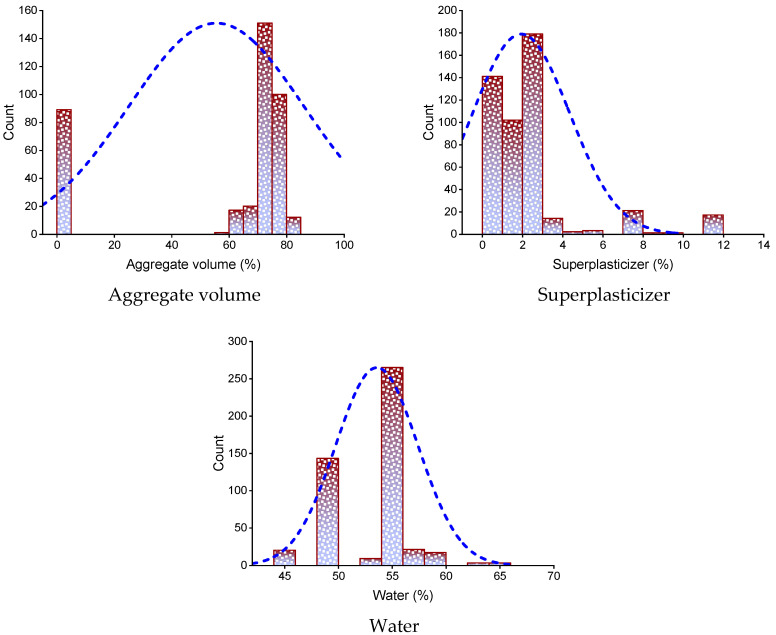
Relative frequency dispersal of inputs parameters.

ML methods need a diverse range of input parameters to acquire the desired outcome [53]. The C-S of GeoPC was forecasted utilizing data obtained from past studies (see Table S1, Supplementary Materials). The data set was arbitrarily selected from the past studies to avoid biased images. This analysis obtained only a C-S-based data set to run the models. The precursor material and activation solution were the same for all data samples, i.e., fly ash and Na_2_SiO_3_–NaOH solution, respectively. Nine input parameters were employed to run the models, including curing temperature, curing time, specimen age, alkali/fly ash ratio, Na_2_SiO_3_/NaOH ratio, NaOH molarity, aggregate volume, superplasticizer, and water, with C-S as the output variable. In the present research, a data set of 481 points was utilized for the outcome prediction using ML methods. The quantity of input parameters and data sets have a considerable impact on the technique’s results [54]. According to prior research, a minimum of 300 data points and eight input variables can result in increased precision for ML models [55,56]. As a result, the data set acquired for this research is optimal for the ML model’s performance. Table 1 lists the descriptive statistical analysis of all input variables. The mode, median, and mean values correspond to central propensity, while the standard deviation, minimum, and maximum values correspond to irregularity. Figure 1 depicts the dispersion of input parameters utilized in the research in terms of their relative incidence. It illustrates the overall number of observations linked to each value or sequence of values.

## 3. Machine Learning Methods Employed

**Figure 2:**
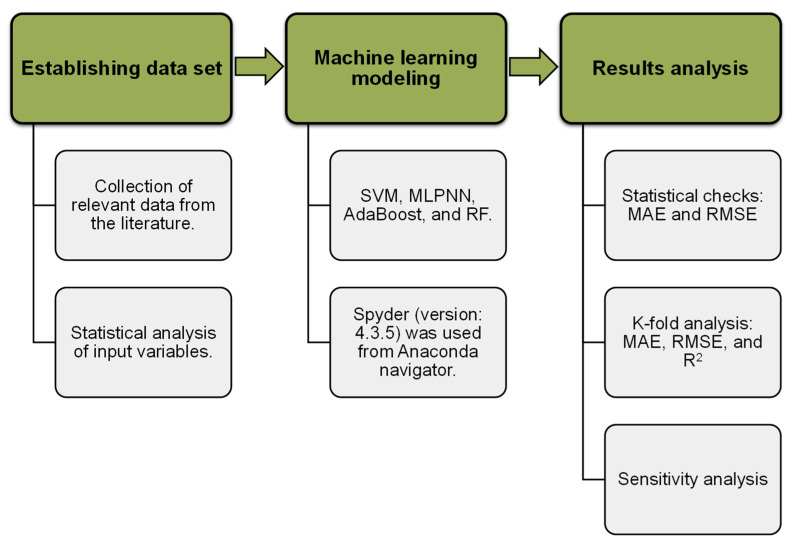
Sequence employed for the study.

Individual ML approaches (SVM and MLPNN), as well as ensemble ML methods (AR and RF), were employed to ascertain the goals of this research with Python codes through the Anaconda Navigator software. Spyder (version 4.3.5) was selected to run the SVM, MLPNN, AR, and RF techniques. These ML methods are typically employed to forecast the required results on the basis of input factors. These methods, amongst other aspects, are able to estimate the temperature influence, the strength characteristics, and the material’s durability [57,58]. The R^2^ value for the expected outcome indicates the performance/validity of ML methods. The R^2^ is a statistic that is used to estimate the degree of variation in a response variable specified by a model. In other words, it quantifies the model’s fit to the data. A value close to zero suggests that fitting the mean is similar to fitting the model, whereas a value near one indicates that the date and model are virtually perfectly suited [59]. The data are split: 20% for testing and 80% for training the ML models. The sub-segments underneath describe the ML approaches used in this study. Furthermore, k-fold evaluation, statistical checks, and error measurements (root mean square error (RMSE) and mean absolute error (MAE)) is performed on all ML methods to validate them. In addition, sensitivity analysis (SA) is carried out to find out the influence of every input variable on the results anticipation. The flow diagram in Figure 2 describes the research technique followed in the present study.

### 3.1. Support Vector Machine

**Figure 3:**
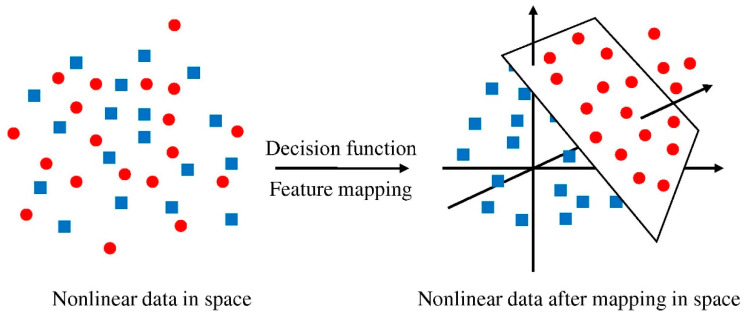
Support vector machine model mapping. Reprinted/adapted with permission from [60].

**Figure 4:**
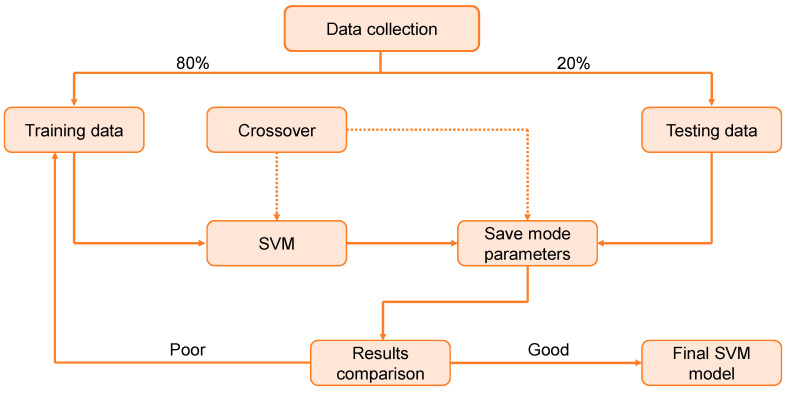
Sequence of support vector machine modeling process.

SVM is an individual ML technique that is used to evaluate data for classification and regression. An SVM technique is a way of describing the samples as points in space that have been plotted in such a way that the patterns of the unique classifications are separated by a distinct vector (line/plane) with the greatest possible separation. Additional cases are then superimposed on that same space and categorized according to which side of the vector they lie on, as illustrated in Figure 3. Figure 4 illustrates the procedure for the SVM model. This model is employed to assess the material’s strength, since it takes into account the combined influence of various components. The optimization approach is used to ascertain the parameters of the SVM model.

### 3.2. Multi-Layer Perceptron Neural Network

**Figure 5:**
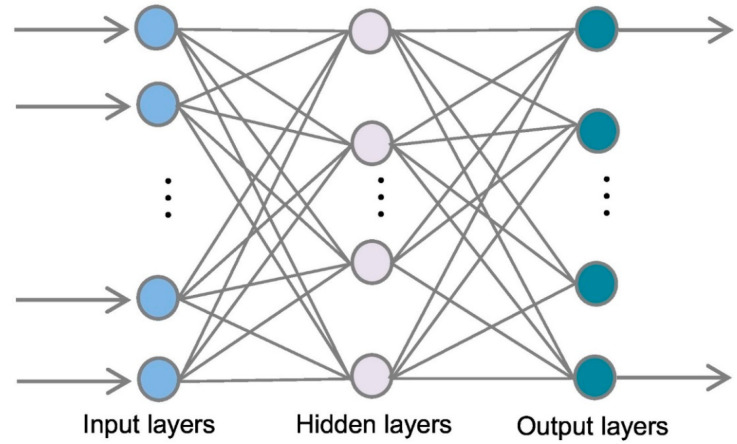
Sequence of a multi-layer perceptron neural network modeling process. Reprinted/adapted with permission from [67].

An artificial neural network (ANN) is a collection of connected nodes that are employed to represent and solve issues that involve complicated interactions among causal events and reactions. MLPNN is one of the highly efficient ANN methods for estimation and modeling. MLPNN has been chosen as the standard method in numerous studies [61,62]. Due to MLPNN’s excellent universal approximation capabilities, it has been commonly utilized to describe nonlinear and complicated phenomena in the actual world [63,64,65]. The MLPNN is a feed-forward technique that comprises a single input layer, one or more hidden layers, and a single output layer [66], as shown in Figure 5. Usually, the number of nodes in the input layer is determined by the data source’s specified factor, while the number of hidden neurons is measured using a particular training data set. The hidden layers are utilized for computing, whereas the output layer is used for modeling. Every node in the hidden layer should be linked to all nodes in the input layer and then to all nodes in the output layer. The MLPNN training operation might split into two steps via these connections: ahead and back, utilizing the back-propagation technique [63].

### 3.3. AdaBoost Regressor

**Figure 6:**
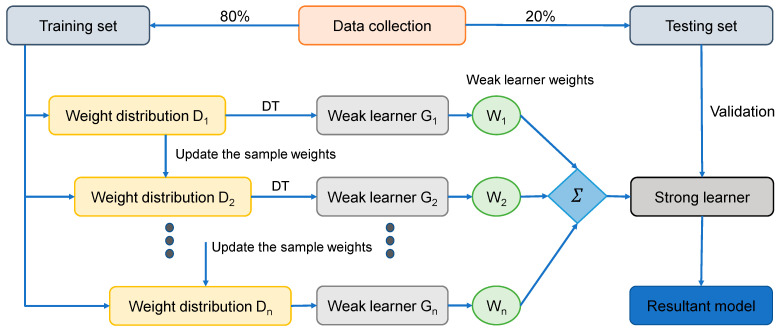
Sequence of AdaBoost regressor modeling process [69].

The AR method is the most common ensemble ML technique in the boosting class. The AR algorithm is unique in that it uses the primary training data to develop a weak learner, and then alters its dispersion of training data based on the projection performance of the weak learner in the subsequent turn of weak learner training. It is important to mention that in the subsequent phase, the training models with lower estimation accuracy from the former phase will receive greater consideration. Following that, the weak learners are combined with a strong learner using a range of weights to create the final pattern [68]. The AR running process is divided into four stages, including data collecting, developing a strong learner, analyzing or confirming the learner, and applying the learner to engineering problems. The second phase is critical to the AR method. As stated before, it is composed of two elements, i.e., a structure for incorporating weak learners into a stronger one and a regression learning algorithm for generating the weak learner from the training data. The SVM technique is employed to construct the weak learner, and the weak learners are combined using the average of the weighted weak learners. The flow diagram for this approach is depicted in Figure 6.

### 3.4. Random Forest

**Figure 7:**
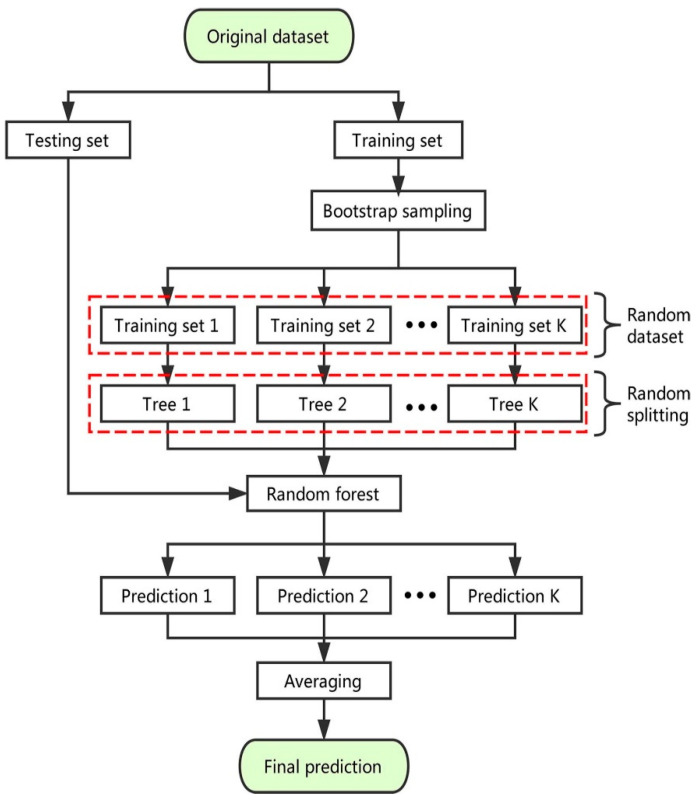
Sequence of random forest modeling process. Reprinted/adapted with permission from [70].

The random split selection technique is used to deploy RF on bagging DTs [70]. Figure 7 schematically depicts the modeling method of the RF technique. Each tree in the forest is produced from an aimlessly selected training set, and every split within a tree is constructed from an erratically chosen subgroup of input parameters, developing a forest [71]. This element of uncertainty increases the tree’s variety. The entire forest is made up of completely mature binary trees. The RF approach has proven to be an extremely powerful tool for general-purpose classification and regression. When the number of variables surpasses the number of observations, the approach, which aggregates the predictions of numerous randomized DTs, demonstrates increased precision. Furthermore, it is adjustable to both large-scale and ad hoc learning tasks, returning measures with varying degrees of significance [72].

## 4. Results and Discussions

### 4.1. Support Vector Machine Model

**Figure 8:**
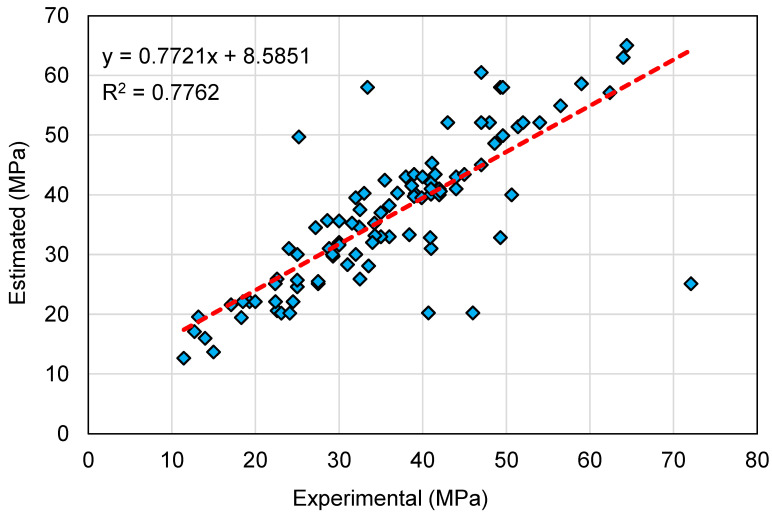
Relationship between experimental and estimated results for the support vector machine model.

**Figure 9:**
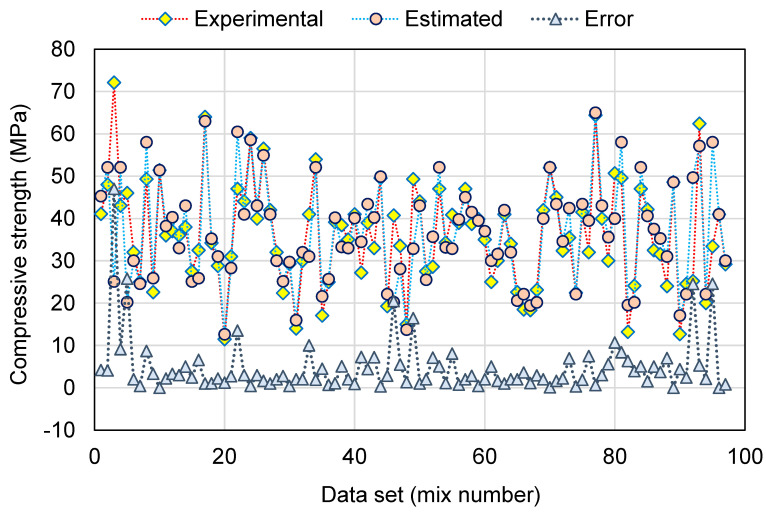
Distribution of experimental, estimated, and error values for the support vector machine model.

The outcomes of the SVM model for the C-S of GeoPC are displayed in Figure 8 and Figure 9. The correlation among the experimental and forecasted results is shown in Figure 8. The SVM method generated results with a lower degree of accuracy and a marginal difference amongst the experimental and forecasted results. The R^2^ of 0.78 confirms that the SVM model has a lower degree of accuracy in anticipating the C-S of GeoPC. Figure 9 demonstrates the dispersion of experimental, anticipated, and error values for the SVM model for testing data alone, which is 20% of the overall data set. The analysis of the experimental and estimated values discovered that the divergence of outcomes (error) was in the limit of 0.00 to 47.0 MPa, with an average of 7.72 MPa. Moreover, for 8 mixes, the divergence from the experimental results was lower than 1 MPa; for 17 mixes, the divergence was between 1 and 3 MPa; for 21 mixes, the divergence was between 3 and 6 MPa; and for 45 mixes, the variance was greater than 6 MPa. This indicated a higher deviation from the projected findings for the SVM model compared to the experimental results. Thus, the SVM technique is less accurate in anticipating the C-S of GeoPC.

### 4.2. Multi-Layer Perceptron Neural Network Model

**Figure 10:**
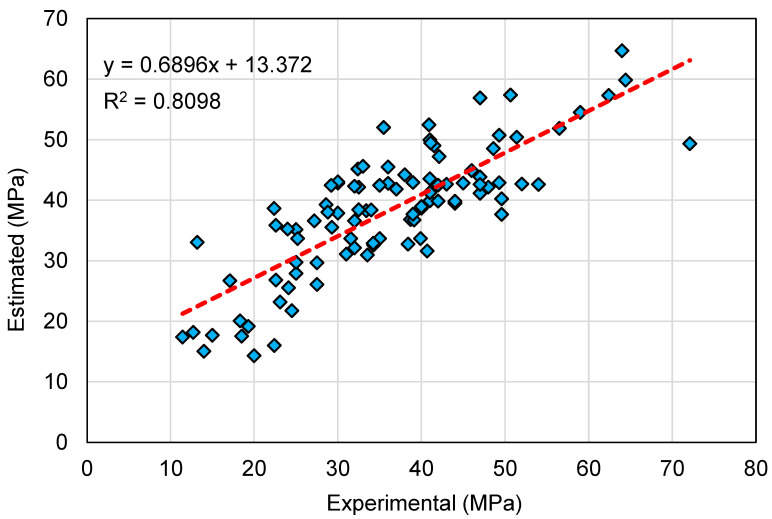
Relationship between experimental and estimated results for the multi-layer perceptron neural network model.

**Figure 11:**
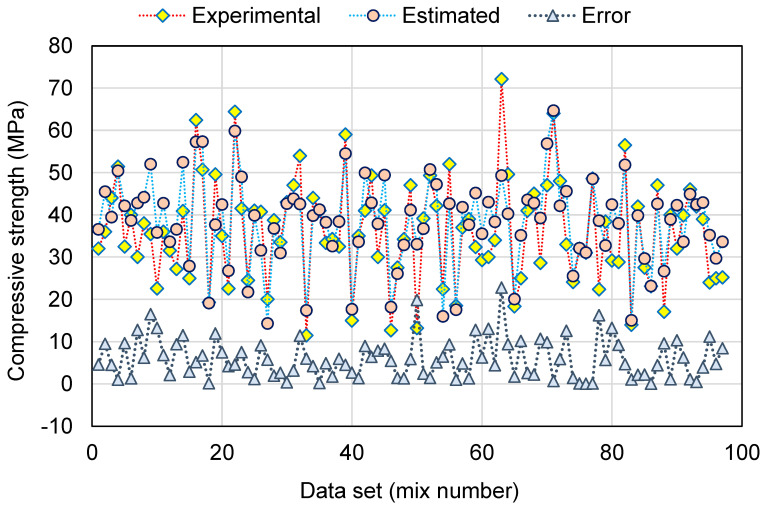
Distribution of experimental, estimated, and error values for the multi-layer perceptron neural network model.

Figure 10 and Figure 11 illustrate a contrast of the MLPNN model’s experimental and anticipated results. Figure 10 exemplifies the relationship among experimental and projected outcomes, with an R^2^ of 0.81 suggesting that the MLPNN model is more specific than the SVM model in estimating the GeoPC C-S. Figure 11 illustrates the distribution of experimental, estimated, and error values for the MLPNN model. The variation between experimental and estimated values was found to be between 0.06 and 22.77 MPa, with an average of 5.86 MPa. Additionally, the variation from the experimental results was lower than 1 MPa for 10 mixes, between 1 and 3 MPa for 25 mixes, between 3 and 6 MPa for 23 mixes, and greater than 6 MPa for 39 mixes. This also indicates a greater divergence of the MLPNN model’s predicted outcomes when compared to the experimental results. Therefore, the MLPNN technique is also less accurate at predicting GeoPC’s C-S, but slightly more accurate than the SVM model.

### 4.3. AdaBoost Regressor Model

**Figure 12:**
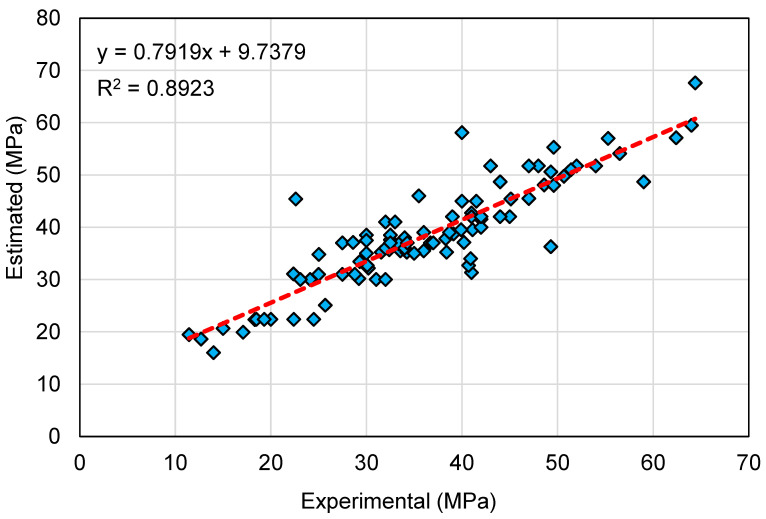
Relationship between experimental and estimated results for the AdaBoost regressor model.

**Figure 13:**
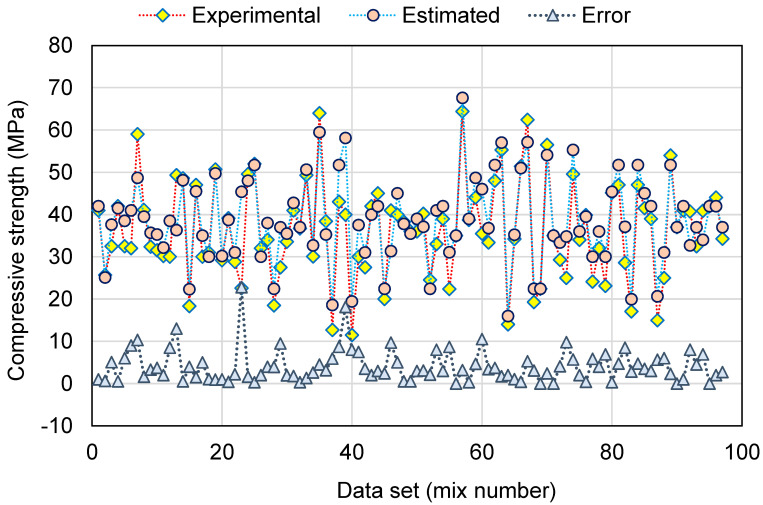
Distribution of experimental, estimated, and error values for the AdaBoost regressor model.

A comparable illustration of the AR model results is depicted in Figure 12 and Figure 13. Figure 12 indicates the relationship among the experimental and anticipated results. The AR method produced outcomes with a higher degree of exactness and a minimal divergence amongst the experimental and projected results. The R^2^ of 0.89 indicates that the AR model is reasonably precise at predicting the C-S of GeoPC. The dispersal of the experimental, anticipated, and error readings for the BR model are shown in Figure 13. The difference (error) between the experimental and estimated values ranged from 0.00 to 22.80 MPa, with a mean of 4.03 MPa. Furthermore, for 18 mixes, the variation from the experimental outcomes was lower than 1 MPa; for 26 mixes, it was between 1 and 3 MPa; for 32 mixes, it was between 3 and 6 MPa; and for only 21 mixes it was larger than 6 MPa. When compared to the experimental data, the AR model’s outcomes showed minimal divergence and higher precision, because this technique uses the training data to build a weak learner and then trains it by altering the dispersal of the training data until it forms a strong learner.

### 4.4. Random Forest Model

**Figure 14:**
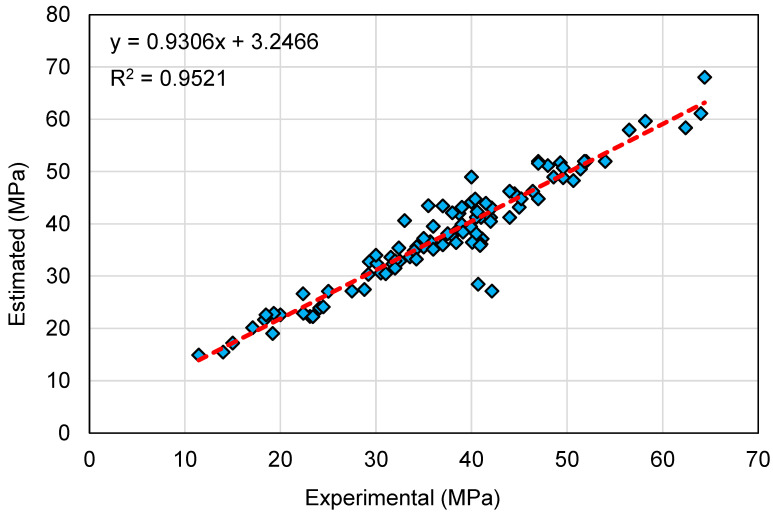
Relationship between experimental and estimated outcomes for the random forest model.

**Figure 15:**
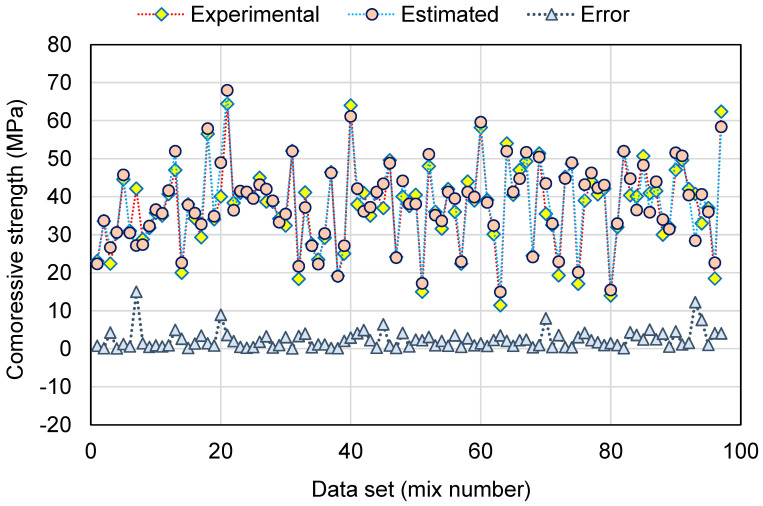
Distribution of experimental, estimated, and error values for the random forest model.

Figure 14 and Figure 15 present a similar representation of the RF model’s results. In Figure 14, an R^2^ value of 0.95 specifies that the RF model performs with the highest precision compared to the other models employed in this study. Figure 15 exemplifies the scattering of experimental, projected, and error values for the RF model. The variation (error) between the experimental and estimated values was found to be between 0.05 and 14.99 MPa, with an average of 2.34 MPa. In addition, the variation from the experimental outcomes was lower than 1 MPa for 38 mixes, between 1 and 3 MPa for 29 mixes, between 3 and 6 MPa for 24 mixes, and greater than 6 MPa for only 6 mixes. This indicates a smaller variation between the experimental and predicted outcomes. Therefore, the RF technique is more suitable, demonstrating the highest precision in estimating the C-S of GeoPC.

## 5. Model’s Validation

K-fold and statistical approaches were employed to validate the performance of all models. Typically, the k-fold analysis method is carried out to find out the model’s validity [73], during which related data are arbitrarily dispersed and split into 10 groups. Nine groups will be utilized for training the models and one will be used for validation. The lower error values (MAE and RMSE) and the higher R^2^ values suggest the higher precision of a model [69]. Moreover, the process must be repeated 10 times to obtain a suitable decision. This broad endeavor provides the notable precision of a model. Moreover, as displayed in Table 2, each ML method was statistically assessed based on errors (MAE and RMSE). These evaluations also supported the ensemble ML model’s greater precision in comparison to the individual techniques, owing to its lower error readings. The projecting accuracy of the models was ascertained statistically through Equations (1) and (2), taken from previous work [55,74,75].

(1) $$\mathrm{MAE}=\frac{1}{n}\overset{n}{\underset{i=1}{\sum }}\left|P_{i}-Ti\right|$$

(2) $$\mathrm{RMSE}=\sqrt{\sum^{\;}\frac{{\left(P_{i}-T_{i}\right)}^{2}}{n}}$$

where *n* = number of data points, *T_i_* = experimental values, and *P_i_* = predicted values.

**Figure 16:**
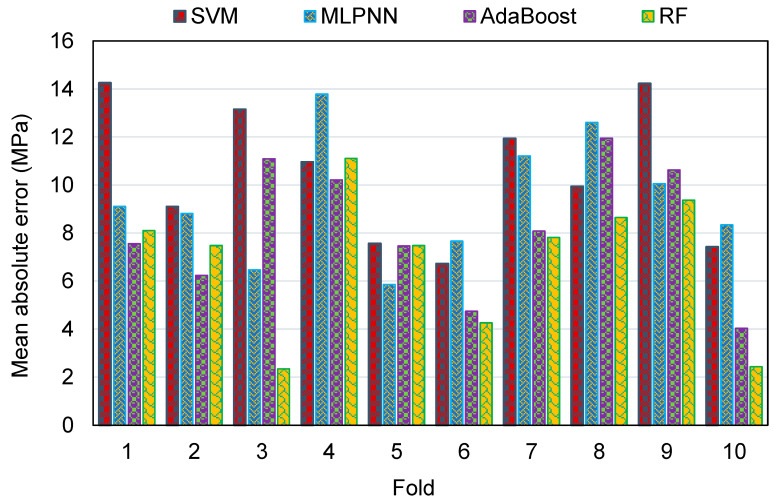
Comparison of mean absolute error for all models from the k-fold analysis.

**Figure 17:**
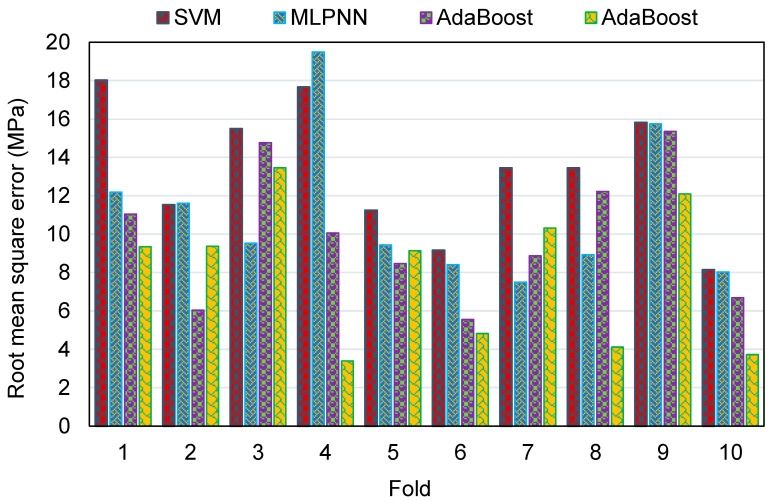
Comparison of root mean square error for all models from the k-fold analysis.

**Figure 18:**
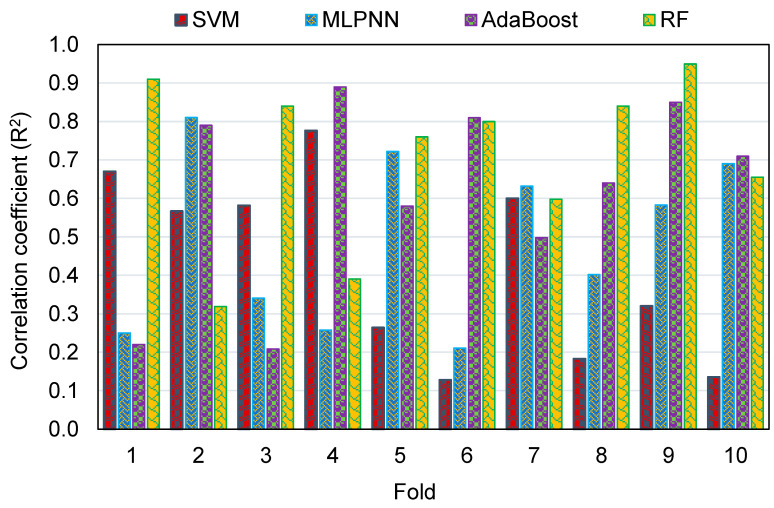
Comparison of the correlation coefficient for all models from the k-fold analysis.

To evaluate the k-fold analysis results, the R^2^, MAE, and RMSE were calculated, and the resulting values for the SVM, MLPNN, AR, and RF techniques are summarized in Table 3. To compare the MAE values for all of the models from the k-fold analysis, Figure 16 was generated. The MAE values for the SVM model were in the range of 6.72 to 14.26 MPa, with an average of 10.53 MPa. The same values for the MLPNN model were between 5.86 and 13.79 MPa, with an average of 9.39 MPa. Additionally, for the AR method, these values were between 4.03 and 11.94 MPa, with an average of 8.20 MPa. The MAE values for the RF model were in the range of 2.34 to 11.10 MPa, with an average of 6.90 MPa. This analysis validated the higher accuracy of ensemble ML models, with the RF model having the lowest error/deviation from the experimental results. This was further confirmed by the results of RMSE, as depicted in Figure 17. The average RMSE value for the SVM, MLPNN, AR, and RF models was 13.29, 11.08, 9.91, and 7.97, respectively. The results of R^2^ from the k-fold analysis were compared and are presented in Figure 18. It was determined that the RF model has higher R^2^ values with an average of 0.71, compared to the other models, which yielded an average R^2^ of 0.42, 0.49, and 0.62 for the SVM, MLPNN, and AR models, respectively. The RF model with smaller deviations from the experimental results and higher R^2^ values outperformed the other models in estimating the C-S of GeoPC. Hence, this analysis suggests the use of an RF model for this purpose.

## 6. Sensitivity Analysis

**Figure 19:**
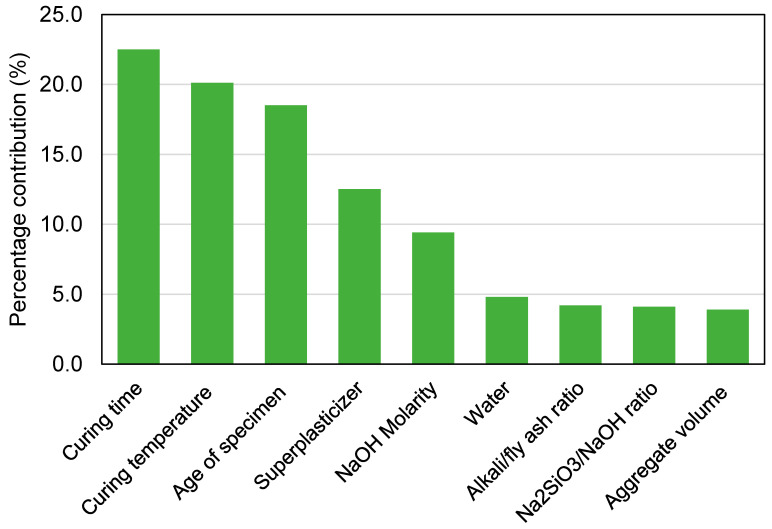
Impact of input factors on the model’s prediction.

The intent of this evaluation is to find out the impact of input variables on GeoPC’s C-S prediction. The anticipated result is considerably influenced by the input factors [76]. Figure 19 illuminates the impact of each input variable on the C-S forecast of GeoPC. The analysis revealed that curing time, curing temperature, and age of specimen were the most important constituents that influence the ML model’s performance in estimating the C-S of GeoPC, accounting for 22.5%, 20.1%, and 18.5%, respectively. The remaining input variables, including superplasticizer, NaOH molarity, water, alkali/fly ash ratio, Na_2_SiO_3_/NaOH ratio, and aggregate volume, had a contribution of 12.5%, 9.4%, 4.8%, 4.2%, 4.1%, and 3.9%, respectively. SA revealed relationships between the quantity of input factors and the data points used to build the ML models. The impact of input parameters on the ML model’s results was ascertained using Equations (3) and (4).

(3) $$N_{i}=f_{max}\left(x_{i}\right)-f_{min}\left(x_{i}\right)$$

(4) $$S_{i}=\frac{N_{i}}{\sum_{j-i}^{n}N_{j}}$$

where $f_{max}\left(x_{i}\right)$ and $f_{min}\left(x_{i}\right)$ are the highest and lowest of the projected outcome over the *i*th output, respectively. The *S_i_* is the attained impact percentage for the specific input parameter.

## 7. Discussions

The objective of this study was to add to the body of knowledge concerning the application of contemporary methods for evaluating the C-S of GeoPC. This kind of exploration will benefit the building industry by facilitating the progress of rapid and cost-efficient material property prediction tools. By encouraging eco-responsive construction through these measures, the adoption and usage of GeoPC in the building sector will be hastened. Since GeoPC might be manufactured from waste constituents, including aluminosilicates, its usage in the building sector has a variety of benefits, including reduced energy consumption, waste reduction, natural resources protection, reduced CO_2_ emissions, better material properties, and green construction materials [27].

This research validates how ML techniques can be utilized to foresee the C-S of GeoPC. Four ML methods were employed: two individual (SVM and MLPNN) and two ensembled (AR and RF). All ML methods were assessed for precision to determine which is the most effective model. The RF model generated a more accurate result with an R^2^ of 0.95, compared to the AR, MLPNN, and SVM models, which yielded R^2^ of 0.89, 0.81, and 0.78, respectively. Furthermore, all models’ performance was confirmed by k-fold and statistical analysis techniques. The fewer errors in the model, the more precise it is. However, establishing and suggesting the ideal ML method for forecasting outcomes across a number of areas is challenging, since any model’s performance is highly dependent on the input parameters and data set utilized to execute the algorithm. Ensembled ML methods frequently make use of the weak learner by building sub-models that may be trained on data and tweaked to maximize the R^2^ value.

**Figure 20:**
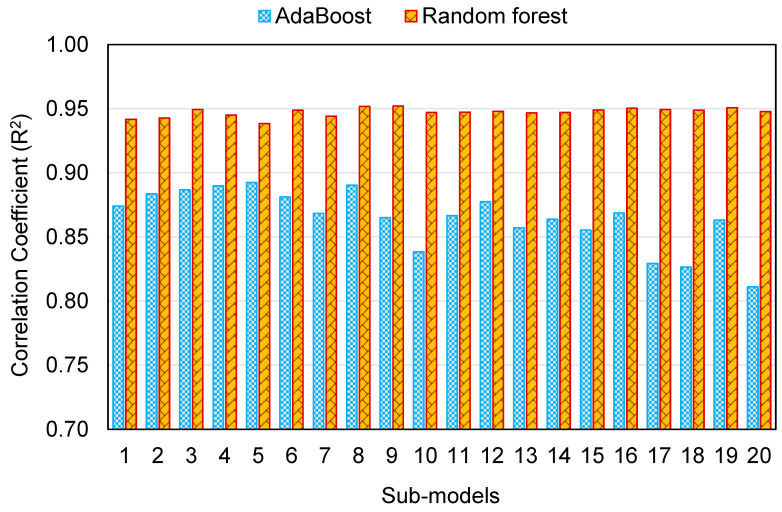
The correlation coefficient of ensemble machine learning sub-models.

The dispersal of R^2^ values for the AR and RF sub-models is represented in Figure 20. The lowest, average, and maximum R^2^ values for AR sub-models were 0.811, 0.864, and 0.892, respectively. The lowest, average, and maximum R^2^ values for RF sub-models were 0.938, 0.947, and 0.952, respectively. These figures indicate the superior exactness of the RF method in comparison to the AR in estimating the C-S of GeoPC. Other researchers have also observed that the AR and RF models are more accurate in predicting outcomes [68,77,78]. Feng et al. [68] observed that the AR model outperformed individual models, including ANN and SVM, in terms of R^2^ and error values. Likewise, Farooq et al. [78] assessed the accuracy of RF with that of the decision tree, gene expression programming, and artificial neural network methods and found that the RF model had a greater precision than the others, with an R^2^ of 0.96.

In addition, an SA was carried out to identify the effect of each input variable on GPC’s anticipated C-S. The model’s effectiveness may be influenced by the input variables and the size of the data set. The SA established the degree to which each of the nine inputs influenced the projected outcome. Curing time, curing temperature, and age of specimen were found to be the three most highly crucial input factors. However, there are several other parameters involved in the manufacture of GeoPC that affect the C-S, such as the chemical composition of the precursors and the superplasticizer, which may be used as input parameters in future ML-based modeling to study their impact. 

## 8. Conclusions

The purpose of this study was to apply both ensemble and individual machine learning (ML) methods to estimate the compressive strength (C-S) of geopolymer concrete (GeoPC). Two individual approaches—support vector machine (SCM) and multi-layer perceptron neural network (MLPNN)—were employed to forecast outcomes, and two ensemble ML approaches were used, namely, AdaBoost regressor (AR) and random forest (RF). This study reached the following findings:

1. Ensemble ML methods (AR and RF) outperformed individual ML techniques (SVM and MLPNN) in forecasting the C-S of GeoPC, with the RF model performing with the highest accuracy. The correlation coefficients (R^2^) were 0.95, 0.89, 0.81, and 0.78 for RF, AR, MLPNN, and SVM models, respectively.
2. The comparison of experimental and anticipated results verified the AR and RF models’ superior accuracy, as the projected values deviated less from the experimental values. On the other hand, the MLPNN and SVM model results deviated more from the experimental results, making them less suitable for predicting the C-S of GeoPC.
3. Statistical analysis and k-fold evaluation were used to validate the model performance. These evaluations validated the RF model’s superior accuracy. The ensembled models’ decreased deviation (MAE and RMSE) and higher R^2^ values supported their increased accuracy over individual models.
4. Sensitivity analysis discovered that curing time, curing temperature, and specimen age were the most significant elements influencing the ML model’s performance in predicting GeoPC’s C-S, accounting for 22.5%, 20.1%, and 18.5%, respectively. The other input variables, including superplasticizer, NaOH molarity, water, alkali/fly ash ratio, Na_2_SiO_3_/NaOH ratio, and aggregate volume, contributed 12.5%, 9.4%, 4.8%, 4.2%, 4.1%, and 3.9%, respectively.
5. This kind of study will benefit the construction industry by allowing for the progress of rapid and cost-efficient strategies for estimating the strength of materials. Moreover, by applying these methods to encourage eco-responsive construction, the acceptance and usage of GeoPC in the building sector will be enhanced.

## Figures and Tables

**Table 1:** Results of the descriptive statistical analysis of input parameters.

Parameter	Curing Temperature (°C)	Curing Time (h)	Age of Specimen (days)	Alkali/Fly Ash Ratio	Na_2_SiO_3_/NaOH Ratio	NaOH Molarity (M)	Aggregate Volume (%)	Superplasticizer (%)	Water (%)
Mean	68.94	27.46	21.53	0.43	2.25	11.89	59.98	1.93	53.56
Median	70.00	24.00	7.00	0.40	2.50	12.00	70.00	1.55	55.90
Mode	60.00	24.00	7.00	0.35	2.50	10.00	70.00	2.00	55.90
Standard Deviation	25.19	13.24	45.33	0.11	0.53	2.73	28.97	2.41	3.82
Range	100.00	92.00	539.00	0.70	3.60	12.00	80.00	11.30	18.90
Minimum	20.00	4.00	1.00	0.30	0.40	8.00	0.00	0.00	45.10
Maximum	120.00	96.00	540.00	1.00	4.00	20.00	80.00	11.30	64.00

**Table 2:** Statistical checks of the ML methods used in the present study.

Model	MAE	RMSE
Support vector machine	6.720	8.145
Multi-layer perceptron neural network	5.864	7.492
AdaBoost regressor	4.027	5.543
Random forest	2.338	3.394

**Table 3:** Results of the k-fold process.

K-Fold	SVM			MLPNN			AR			RF		
	MAE	RMSE	R^2^	MAE	RMSE	R^2^	MAE	RMSE	R^2^	MAE	RMSE	R^2^
1	14.26	18.02	0.67	9.10	12.19	0.25	7.56	11.05	0.22	8.10	9.34	0.91
2	9.09	11.53	0.57	8.81	11.62	0.81	6.23	6.04	0.79	7.48	9.36	0.32
3	13.15	15.48	0.58	6.46	9.53	0.34	11.09	14.76	0.21	2.34	13.46	0.84
4	10.96	17.65	0.78	13.79	19.49	0.26	10.21	10.06	0.89	11.10	3.39	0.39
5	7.56	11.24	0.26	5.86	9.43	0.72	7.46	8.46	0.58	7.49	9.13	0.76
6	6.72	9.16	0.13	7.67	8.40	0.21	4.74	5.54	0.81	4.26	4.81	0.80
7	11.94	13.45	0.60	11.21	7.49	0.63	8.08	8.87	0.50	7.81	10.31	0.60
8	9.94	13.45	0.18	12.60	8.92	0.40	11.94	12.23	0.64	8.65	4.11	0.84
9	14.23	15.82	0.32	10.05	15.75	0.58	10.62	15.35	0.85	9.38	12.10	0.95
10	7.43	8.15	0.14	8.34	8.03	0.69	4.03	6.69	0.71	2.44	3.72	0.66

## Data Availability

The data used in this research have been properly cited and reported in the main text.

## Supplementary Materials

The following supporting information can be downloaded at: https://www.mdpi.com/article/10.3390/polym14102128/s1, Table S1: Data used for modeling.
